# Caregiver Satisfaction with a Video Telehealth Home Safety Evaluation for Dementia

**DOI:** 10.5195/ijt.2020.6337

**Published:** 2020-12-08

**Authors:** Megan E. Gately, Linda Tickle-degnen, Scott A. Trudeau, Nathan Ward, Keren Ladin, Lauren R. Moo

**Affiliations:** 1 Geriatric Research Education and Clinical Center, VA Bedford Healthcare System, Bedford, Massachusetts, United States; 2 Department of Occupational Therapy, Tufts University, Medford, Massachusetts, United States; 3 Department of Psychology, Tufts University, Medford, Massachusetts, United States; 4 American Occupational Therapy Association, Bethesda, Maryland, United States; 5 Department of Community Health, Tufts University, Medford, Massachusetts, United States; 6 Department of Neurology, Harvard Medical School, Boston, Massachusetts, United States

**Keywords:** Caregivers, Dementia, Safety, Telemedicine

## Abstract

Family caregivers are vital to telehealth-delivered dementia care. The objective of this mixed methods descriptive study conducted in the VA Bedford Healthcare System was to examine caregiver satisfaction with a video telehealth dementia home safety occupational therapy evaluation. Ten caregivers of Veterans with dementia participated. Ratings of caregiver satisfaction, measured by nine Likert scale items including ability to see and hear, were examined in relation to person and visit-related contextual factors extracted from research assistants' field notes, to develop an in-depth understanding of caregiver experience. Person factors included caregiver age and gender and Veteran cognitive status. Visit-related contextual factors included occurrence of technical glitches. Caregiver visit satisfaction was overall positive, with exceptions related to technological glitches and the presence of the person with dementia during the visit. Veteran cognitive status appeared to influence caregiver satisfaction. Implications of the study are that proactively addressing technical glitches and incorporating dementia stage-specific approaches may optimize caregivers' telehealth experience.

Individuals with dementia experience progressive cognitive decline affecting judgment, communication, emotional, psychological, and motor functioning. These changes lead to increased and evolving safety risks within the home environment, necessitating home modifications. Home safety evaluations for dementia are performed in the context of progressive cognitive deficits that affect individual functioning. As such, they are ideally client-centered, reflecting complex person-environment-occupation factors affecting individuals with dementia and their caregivers (Struckmeyer & Pickens, 2016).

The Person-Environment-Occupation (PEO) model highlights that everyday functioning is the result of optimal interaction between the person, the environment, and the occupation (target tasks). PEO describes these overlapping, inextricably linked domains which are involved with day-to-day functioning. The person domain embodies individuals' roles, identities, and health status. The environment includes physical structures and sociocultural factors, such as the social network. Occupation refers to the tasks a person wants and needs to do. Imbalance in any one domain reduces occupational performance or day-to-day functioning (Law et al., 1996). This model emphasizes the fit between the individual and their preferred activities within the home setting to optimize functional performance. Adjustments to any of the domains of PEO can influence both safety and functional performance.

For individuals with dementia, caregivers are central to this PEO transaction. Caregivers increasingly speak for the person with dementia, and are influential to their health and well-being, particularly as cognitive status worsens. Similarly, the level of home safety risk due to dementia behaviors such as wandering, and the degree to which caregivers are confident in promoting home safety, influence the relative success of home safety strategies (Horvath et al., 2005). All of these complex PEO factors are relevant to delivery of dementia home safety evaluations. Though occupational therapist-led home safety evaluations are the gold standard (CDC, 2019; Maggi et al., 2018; Pighills et al., 2016), in that occupational therapy (OT) practitioners are trained to consider the complex interaction of PEO factors, OT-led home safety evaluations are often not available (Lin et al., 2015).

Video telehealth is care in which patient and provider are in two locations synchronously connected via videoconferencing. Video telehealth may increase access to dementia care; however, in-home video telehealth for dementia is undeveloped. Our scoping review of in-home video telehealth identified primarily time-limited, protocolized caregiver support programs (Gately et al., 2019). No study included a home safety evaluation, which is a complex intervention involving a room-by-room assessment. Though video telehealth has been employed for home safety (Renda, 2018; Sanford et al., 2009), no study has relied on a caregiver of a person with dementia to operate technology in the home. This study aims to address this gap by examining caregiver satisfaction of a video telehealth delivered home safety evaluation in relation to PEO factors.

To directly link the PEO model to caregivers' satisfaction of a video telehealth home safety evaluation, we draw from the work of Lee and Coughlin (2015) about technology adoption by older adults. They identified interrelated factors—Individual, Social, Technology, and Delivery—found to influence older adults' adoption of technological solutions and strategies. Their Individual and Social factors represent in our study, Person characteristics of the caregiver and care recipient dyad that are relevant to technology adoption. In our study, their Technology factor represents the Environmental and Occupational characteristics relevant to the video telehealth home safety intervention, including use of technology. The Delivery factor represents broader health care delivery, including clinician and organizational factors that impinge upon or promote successful adoption of technological innovations like video telehealth. For our study the PEO model guided our selection and description of our study measures, and Lee and Coughlin's model guided suggested adaptations to video telehealth home safety evaluations, based on our findings (Lee & Coughlin, 2015).

## METHODS

This mixed methods descriptive analysis involves data drawn from a previously published study of video telehealth dementia home safety evaluations provided to caregivers of Veterans with dementia. The primary aims of the initial study were to examine feasibility of the telehealth encounter. Complete study details are reported elsewhere (Gately et al., 2020). Here we examine caregiver satisfaction with the video telehealth home safety evaluation, through in-depth examination of caregiver satisfaction relative to person, environment, and occupation (PEO) factors. The study was approved by VA Bedford Healthcare System Institutional Review Board.

## PARTICIPANTS

A convenience sample of self-identified family caregivers of community-dwelling Veterans with dementia with a scheduled visit at either the in-person or video telehealth dementia management clinics at VA Bedford Healthcare System participated. Caregivers could embody one of a variety of roles (e.g., spouses, adult children, friend) and were not required to live with the Veteran. Given the cognitive and physical demands of the telehealth evaluation (which involved navigating the home while holding a portable computing device under clinician instructions), caregivers, rather than Veterans with dementia, were study participants. Caregivers needed to be English-speaking, have basic computer skills (e.g., ability to email), normal or corrected-to-normal vision and hearing, and adequate mobility to navigate the home, which was determined through self-report during the informed consent process once the study procedures were described. There were no other inclusion or exclusion criteria.

## DESCRIPTION OF THE INTERVENTION

Prior to the video telehealth home safety evaluation, caregivers received a standard, in-person, non-technological evaluation to expose caregivers to a typical home evaluation. Trained graduate-level research assistants (RAs) administered both types of evaluations under supervision of the principal investigator (PI). In-person and video evaluations followed similar procedures: a brief interview to ascertain home safety concerns followed by room-by-room assessment using a checklist (“Worksheet for making the home safer for a person with memory loss,” 2019). The checklist included items related to whether walkways and stairs were free of clutter and presence of firearms, for example. For the video telehealth home safety evaluations, caregivers navigated the home while holding a laptop or tablet under verbal direction of the study staff (PI-RARA) based at the hospital. During the evaluation, caregivers went up and down stairs and outside the home, as appropriate and feasible, under remote RA instruction. Study staff monitored participants for adverse events or unforeseen hazards during procedures and were prepared to assist and/or notify emergency responders as needed. Video telehealth home evaluations were conducted approximately four days after in-person home evaluations.

## OUTCOME MEASURE

### CAREGIVER SATISFACTION

We employed an investigator-developed, nine-item visit satisfaction questionnaire utilized by co-author, Moo, in her study of in-home video telehealth for dementia (Moo et al., 2014). Items gathered information about caregivers' ability to see, hear, communicate with, and understand the provider; their comfort using technology and whether there was enough technical assistance; whether the visit was sufficiently private; whether the visit was an efficient use of time; and their visit format preference. The questionnaire employed a five-point Likert scale of agreement ranging from Strongly Disagree (1) to Strongly Agree (5). For all items, stronger agreement indicated a more positive experience. Caregivers completed the questionnaire over video telehealth or phone with the PI (without the RA present) immediately following video telehealth home safety evaluations.

#### PERSON, ENVIRONMENT, OCCUPATION FACTORS

See Table 1 for a list of study variables by PEO factor. Caregiver age, gender, race, and relationship to the Veteran, were gathered via standard demographic questionnaire. Veteran factors, including gender, age, racial and ethnic self-designations, and cognitive status, which was represented by most recent Mini-Mental Status Examination (MMSE) score, were gathered via chart review. MMSE scores to indicate dementia stage included 20 to 24 for mild dementia, 13 to 20 for moderate dementia, and less than 12 indicating severe dementia (Folstein, 1975).

**Table 1:** Summary of Variables by PEO Domain

Variable	Source	PEO domain
Caregiver demographics, e.g., age, gender	Demographic questionnaire	P, E
Veteran demographics, e.g., age, cognitive status	Chart review	P
Veteran risky behaviors	Risky Behaviors Questionnaire	P, E
Caregiver confidence in home safety	Confidence in Caregiving Scale	P, E
Visit details of the home safety evaluation	Field notes	E
Caregiver satisfaction of virtual home evaluation	Satisfaction questionnaire	P, E, O

*Note*: Please see Methods for when each variable was gathered. Abbreviations: P, person. E, environment. O, occupation.

Dementia-specific person factors included Veteran dementia risky behaviors and caregiver confidence in addressing home safety in dementia, which were gathered at baseline using two standardized measures: Risky Behaviors Questionnaire and Confidence in Caregiving Scale.

The Risky Behaviors Questionnaire is a one-page, 22-item checklist developed for use in the clinical trial of a dementia Home Safety Toolkit (Horvath et al., 2013). This questionnaire has demonstrated content validity and gathers behaviors common in dementia, such as instances of wandering and sleep disturbance. Designed to capture data at baseline and biweekly for three months, the outcome is the summed total of risky behaviors that occurred. Individual behaviors are not weighted because it is difficult to determine the severity of an incident. Thus, potential scores on the questionnaire range from 0 to undetermined with an indeterminate maximum score. For this study, caregivers were asked at baseline to provide the total number of Veteran Risky Behaviors in the prior month.

The Confidence in Caregiving Scale includes the 12-item Home Safety sub-scale, created by researchers at VA Bedford Healthcare System (Horvath et al., 2013). The Home Safety sub-scale asks caregivers to rate on a scale of 0-100 their perceived confidence in preventing dementia home safety behaviors such as wandering and eating non-food items.

RAs also completed field notes immediately following evaluations. Field notes included brief descriptions of who was present during evaluations, any technological difficulties, and other perceived challenges.

## DATA ANALYSIS

Veteran and caregiver demographics, risky behaviors, caregiver confidence, and caregiver visit satisfaction scores were examined and summarized using descriptive statistics. Total scores or individual item scores were examined participant-by-participant to explore systematic variation among variables. Specifically, caregiver visit satisfaction scores on each item were compared with caregiver and Veteran demographic factors, risky behaviors, and caregiver confidence. Caregiver satisfaction scores were ordered from lowest to highest by risky behavior scores and Veteran variables such as age and cognitive status. Any similarities, differences, and patterns between satisfaction scores and person factors such as caregiver age and Veteran risky behaviors were noted. RA field notes were analyzed using conventional content analysis (Hsieh & Shannon, 2005) whereby visit notes were repeatedly read by the PI with the sole purpose of helping to explain the few instances of lower caregiver satisfaction.

## RESULTS

### PARTICIPANT CHARACTERISTICS

Table 2 shows Veteran-caregiver characteristics ranked by Veteran's MMSE score which ranged from 3 to 22 (out of 30). Six caregivers were spouses and four adult children. Caregiver age ranged from 54 to 71 (average 62.8 years old). Eight caregivers were female and two were male. Most Veterans (90%) were male. All but two caregivers lived with the Veteran. Self-reported caregiving hours ranged from eight to 133 hours per week, with most caregivers (60%) providing over 100 hours of caregiving per week, while caregiving duration ranged from 18 to 144 months. Veteran cognitive status varied, with most Veterans (60%) in the mild-to-moderate stages of dementia, as indicated by MMSE score >12. Most caregivers (90%) reported at least one Veteran risky behavior in the month prior to enrollment (range 0 to 81).

**Table 2:** Summary of Caregiver and Veteran Characteristics

ID	Veteran MMSE	Veteran Age	Veteran ^1^Risky Behaviors (Total)	Care-giver Age	Care-giving Hours/Week	Care-giving Duration (Months)	Caregiver ^2^Confidence in Home Safety	Caregiver Gender	Caregiver Role
*2*	3	69	38	67	133	120	98%	Female	Partner
*7*	9	70	14	63	120	18	80%	Female	Partner
*5*	9	67	81	62	114	50	93%	Female	Partner
*3*	11	90	20	57	120	96	86%	Male	Child
*10*	15	84	26	67	64	120	97%	Female	Partner
*6*	16	85	4	59	8	36	99%	Male	Child
*4*	17	83	16	54	48	96	56%	Female	Child
*1*	17	84	19	71	116	144	83%	Female	Partner
*8*	20	82	0	58	15	42	68%	Female	Child
*9*	22	74	26	70	133	24	91%	Female	Partner
*Median* 15.5	82.5	19.5	62.5	87.1	73	88.5			

*Note.* Variables sorted by Veteran MMSE score. Identification numbers listed are from our manuscript related to technical feasibility (Gately et al., 2020).

1 Total number of Veteran risky behaviors, e.g., instances of wandering, falls, etc., in the past month.

2 Average of 12-items on Home Safety Confidence in Caregiving sub-scale. Abbreviations: MMSE, Mini-Mental Status Examination.

#### CAREGIVER SATISFACTION

Table 3 shows caregiver satisfaction ratings. Most caregivers (80%) rated less than strong agreement (<5) on one or more visit satisfaction questions. However, low ratings were few, with all caregivers either strongly agreeing, agreeing, or being neutral about visit satisfaction for the following items: ease of communicating with the provider (*Median* = 4, range 3-5); ability to understand provider (*Median* = 5, range 4-5); comfort with technology (*Median* = 5, range 4-5); and, enough technological assistance (*Median* = 4, range 3-5). The only person factor that appeared to relate to caregiver satisfaction was Veteran MMSE, in that caregivers of Veterans with severe dementia (MMSE <12) were more often satisfied (*Median* across all items was 5 for three of four caregivers in this group, range 3-5) compared to caregivers of Veterans with mild-to-moderate dementia (*Median* across all items was 4 for five of six caregivers in this group, range 1-5). This pattern was observed even though Veterans with severe dementia had more risky behaviors than Veterans with mild-to-moderate dementia; Veterans with severe dementia (MMSE <12) had an average 38.3 risky behaviors (range 14-81) compared to those with mild-to-moderate dementia, who had an average 15.2 risky behaviors (range 0-26)

**Table 3:** Caregiver Satisfaction with Video Telehealth Home Safety Evaluation

ID	Veteran MMSE	Able to Hear	Able to See	Easy to Communicate	Understood Provider	Visit Private Enough	Comfort Using Tech	Enough Technical Assistance	Visit Efficient Use of Time	Prefer Video to In-Person	Caregiver Score Across Items (*Median*)
*2*	3	3	3	3	4	4	4	4	5	3	4
*7*	9	5	5	4	5	5	5	4	5	3	5
*5*	9	5	5	5	5	5	5	5	5	3	5
*3*	11	5	5	5	5	5	—	—	5	3	5
*10*	15	5	2	5	5	5	5	5	5	3	5
*6*	16	4	4	5	5	4	5	3	5	3	4
*4*	17	5	4	4	5	1	5	4	4	1	4
*1*	17	4	4	4	4	4	4	4	4	2	4
*8*	20	2	4	3	4	5	4	3	4	3	4
*9*	22	4	4	4	4	4	4	5	2	2	4
*Median*		4.5	4	4	5	4.5	5	4	5	3	4

*Note.* Caregiver responses to visit satisfaction questionnaire. Responses were in five-point Likert scales of agreement ranging from Strongly Disagree (1) to Strongly Agree (5). All variables sorted by Veteran MMSE. Identification numbers listed are from our manuscript relate to technical feasibility (Gately et al., 2020).

Three of the four instances of lower caregiver satisfaction (disagree or strongly disagree) for caregivers 8, 9, and 10 appeared to relate to the environmental factor of technological glitches. In the one report of difficulty hearing the provider (caregiver 8), field notes indicated the visit began with eight minutes of technical assistance for audio difficulty, as “speakers on RA computer were not turned on.” In the single report of difficulty seeing the provider (caregiver 10), field notes indicated the caregiver's screen froze towards the end of the visit and that “CG reported she did not have view of RA for about 15 minutes.” The RA chose to continue the visit, however, since she was still able to see the caregiver and the home. Similarly, in the single report of the visit not being an efficient use of time (caregiver 9), field notes mentioned 70 minutes of technical assistance, which included caregiver difficulty logging in. Field notes indicated that the process of trouble-shooting “took a little over an hour until RA was able to call CG successfully and connect, but with no audio,” at which point the caregiver opted to use a laptop for video and a phone for audio. Details of all technological glitches encountered are reported elsewhere (Gately et al., 2020).

For the remaining instance of strong disagreement, the presence of the person with dementia during the home safety evaluation appeared to influence caregiver satisfaction. In the single report of the evaluation not being private enough (caregiver 4), field notes revealed that the Veteran was shadowing the caregiver (accompanying her to each area of the home during the evaluation) and appeared “annoyed and confused by the purpose of the visit.” Of note, this evaluation occurred later in the day, when increased confusion in people with dementia is common. This caregiver also strongly disagreed with preferring the video telehealth evaluation.

## DISCUSSION

To our knowledge, this is the first study to examine satisfaction with video telehealth delivered dementia-focused home safety evaluations, specifically employing a caregiver of the person with dementia to operate technology. Actively involving caregivers of persons with dementia in video telehealth aligns with recent evidence highlighting the importance of caregiver and family-centered models in dementia technological approaches (Sriram et al., 2019). Since most persons with dementia live in the community, engaging family caregivers to assist with telehealth may increase access to care when sending paid care staff into the home is not feasible. It is also in accordance with the need for dementia technologies that reduce risk and prevent negative outcomes (Astell et al., 2019).

Returning to PEO, we found that the person factor of cognitive status of the person with dementia appeared to influence caregiver satisfaction ratings, as did the environmental factors of the presence of the person with dementia and technological glitches. Below are key findings and strategies to optimize caregiver satisfaction, organized by Lee and Coughlin's (2015) technology adoption factors: Individual and Social, Technology, and Delivery. Similar to PEO, these factors structure potential ways to optimize video telehealth.

### INDIVIDUAL AND SOCIAL

Overall positive caregiver satisfaction across caregiver age, role, gender, and caregiving duration, including substantial caregiving hours, suggests that in-home video telehealth may be appropriate for caregivers with a variety of PEO profiles. This aligns with prior work indicating openness of caregivers of persons with dementia to technological strategies (Lindauer et al., 2017). The finding that greater satisfaction occurred in caregivers of Veterans with more severe dementia despite there being more Veteran risky behaviors, may indicate caregiver resilience as dementia progresses (Harris, 2008). It may also suggest that caregivers are more willing to endure technological glitches of a virtual home safety evaluation when there is greater perceived home safety risk. Given indications that implementing behavioral modifications in the early stages may increase carry-over into later stages (Harris, 2002), we recommend intervening early in dementia to minimize home safety risk.

High caregiver satisfaction in communication domains underscores the importance of clear, effective verbal communication, which is even more pronounced in a home safety evaluation than in other less mobile video telehealth. We found the process of a video telehealth home safety evaluation required constant cueing and directing (e.g., tilt the camera, pan more slowly) to better see the home for accurate assessment (Gately et al., 2020). This process occurred while caregivers were physically walking around the home and holding remote computing devices, a highly complicated process. Balancing the need to maximize visualization of the environment with not overly stressing caregivers by bombarding them with directions may increase caregiver satisfaction.

### TECHNOLOGY

The negative impact of technological glitches on caregiver satisfaction aligns with research highlighting problems with technology as a barrier to technology for older adults (Vaportzis et al., 2017). Caring for someone with dementia is demanding which may amplify the negative impact of technological glitches. This makes it more important to proactively address or anticipate technological needs by providing training (Waller et al., 2017) and conducting a test session ahead of time. Having technical support available may optimize a caregiver's experience. Since user-friendly, easy-to-use technology increases older adults' willingness to utilize technology (Kerssens et al., 2015; Wang et al., 2019), allowing caregivers to use their own technological devices may soften the learning curve.

### DELIVERY

Our findings suggest several considerations for health care systems planning to deliver in-home video telehealth home safety evaluations for dementia. Timing the evaluation to avoid behavioral disturbances such as commonly occur later in the day may optimize the experience for both caregiver and person with dementia. Also, for our study, caregivers were consumed with operating the technology and thus were unable to monitor or supervise the person with dementia. This may result in safety concerns if the person with dementia cannot be left unattended. This highlights the potential need for contingency planning, (e.g., having another person present in the home).

## STUDY LIMITATIONS

This study had several limitations, including a small convenience sample, participants' racial and ethnic homogeneity, and the fact that Veterans with dementia were mostly male. Larger studies with persons with dementia and caregivers from more diverse racial and ethnic backgrounds will broaden our understanding of the relationship between PEO factors and experience of telehealth. In terms of methodology, we have limited qualitative data, and due to sample size, cannot demonstrate statistical significance. The PI, who was present during the evaluation, administered the caregiver satisfaction scale, introducing possible response bias. We also did not ask Veterans for their visit satisfaction, whereas persons with dementia express interest in being included in technological studies (Meiland et al., 2017).

## IMPLICATIONS FOR OCCUPATIONAL THERAPY PRACTICE

Our study suggests the following implications for video telehealth home safety evaluations for dementia:

- Prepare before the visit by offering the caregiver a technology trial and discuss possible contingencies such as having another person present to occupy the individual with dementia during the visit.
- Effective communication is paramount, given the nature of technology-mediated communication and the dynamic nature of the intervention.
- Streamline the technological experience by training caregivers ahead of time and ensuring technical support is available.
- Consider visit timing and potential safety concerns for the person with dementia when relying on caregivers to operate technology.

## CONCLUSION

Recognition of person-environment-occupation factors will ensure client-centered video telehealth that is well-received by populations contending with even the most complex chronic conditions. We found the detection of patterns in home safety evaluation caregiver satisfaction was expedited by employing the PEO framework. This framework can be utilized in larger, more controlled studies of video telehealth.
